# The 2014 Nobel Prize in Physiology or Medicine: A Spatial Model for Cognitive Neuroscience

**DOI:** 10.1016/j.neuron.2014.12.009

**Published:** 2014-12-17

**Authors:** Neil Burgess

**Affiliations:** 1 Institute of Cognitive Neuroscience and Institute of Neurology, University College London, 17 Queen Square, London WC1N 3AR, UK

## Abstract

Understanding how the cognitive functions of the brain arise from its basic physiological components has been an enticing final frontier in science for thousands of years. The Nobel Prize in Physiology or Medicine 2014 was awarded one half to John O’Keefe, the other half jointly to May-Britt Moser and Edvard I. Moser “for their discoveries of cells that constitute a positioning system in the brain.” This prize recognizes both a paradigm shift in the study of cognitive neuroscience, and some of the amazing insights that have followed from it concerning how the world is represented within the brain.

## Teaser

The 2014 Nobel Prize in Physiology or Medicine was awarded to John O’Keefe, May-Britt Moser, and Edvard I. Moser “for their discoveries of cells that constitute a positioning system in the brain.” Here Burgess discusses their work, its influence, and future implications.

## Main Text

### Introduction

Early ideas concerning the production of thought were varied, unconstrained by direct experimental data, e.g., Hippocrates’ theory of cognition depending on the balance of the four humors (blood, phlegm, and two types of bile). However, with the advance of technology, the importance of electrical signaling in the brain and nervous system slowly became accepted, from the effects of electrical stimulation on muscles noted by Luigi Galvani in the 18th century, to the cognitive effects in the human brain noted by Wilder Penfield in the 1940s and 50s. The realization of the importance of neurons (the “neuron doctrine”) by Santiago Ramon y Cajal—recipient of the Nobel Prize in 1906 with Camillo Golgi—combined with an appreciation of the role of electricity gave rise to the modern field of electrophysiology as a tool to understand brain function. Early successes included an understanding of neural responses to light in the retina, for which Granit, Hartline, and Wald received the Nobel Prize in 1967, leading to an understanding of the feedforward processing of simple visual stimuli by neurons in the visual system of anaesthetised mammals for which David Hubel and Torsten Wiesel received the Nobel Prize in 1981.

Amidst these exciting developments in the 1960s, John O’Keefe was studying his PhD at McGill University, receiving instruction from, among others, Donald Hebb—one of the foremost proponents of looking for direct analogs of learning and experience within the brain. As he began his research career, O’Keefe was persuaded of the “neuro-ethological” approach in which, faced by a lack of knowledge about how the brain works, the primary aim is a broad survey of how the system in question is used during normal behavior. This approach contrasts with the principles of more traditional experimental design, with its focus on tightly controlled testing of well-specified hypotheses, and is well described in the book written later with Lynn Nadel, his friend and colleague from their time at McGill, *The Hippocampus as a Cognitive Map* (O’Keefe and Nadel, 1978).

O’Keefe also profited from the timely advent of modern transistors, allowing robust differential recording of electrical activity from freely behaving animals, in which the large variations in potential common to nearby electrodes could be cancelled and the remaining signals boosted on the animal’s head. Along with other early pioneers such as Jim Ranck, this allowed O'Keefe to introduce a new paradigm in understanding brain function—studying neuronal activity in freely behaving animals in response to naturalistic stimuli or tasks. This paradigm has continued to spread, slowly, over the subsequent decades, notwithstanding the intrinsic validity of more traditional approaches in narrowing down potential functions as each function becomes more completely understood. With the retrospect of the subsequent 40 years of progress, the neuro-ethological approach of electrophysiology in freely behaving animals appears to be a crucial “paradigm shift” for cognitive neuroscience of the kind identified by Thomas Kuhn.

Two of the most dramatic results of the neuro-ethological approach to electrophysiology are honored by the Nobel Prize: the discoveries of place cells and of grid cells. As noted in the Nobel citation, the properties of these cells, along with those of other types of spatial cell, most notably head direction cells, help to define “a positioning system in the brain.” Identification of this specific system is indeed a great achievement in understanding the link between mind and brain. But perhaps even more important are the general lessons they provide for the neural coding of internal cognitive constructs, and the neural mechanisms of learning, representation, and memory.

### Place Cells and Cognitive Maps

In his first experiments, published with Herman Bouma in 1969, O’Keefe recorded responses in the amygdala of freely moving cats to ethologically relevant stimuli (birdsong, mouse shapes, etc.). They found many instances of neurons that appeared to be tuned to represent the presence of specific stimuli, when presented, and some that continued to respond after the stimulus was withdrawn. These findings have been echoed recently by neurons in human amygdala that respond to pictures of specific animals.

Both the amygdala and the neighboring hippocampus had already been implicated in human memory by the finding, at the nearby Montreal Neurological Institute founded by Penfield, that bilateral damage or removal of these structures and surrounding cortical tissue in the treatment of epilepsy caused profound amnesia (Scoville and Milner, 1957). This work immediately revised the preceding view of the hippocampus as part of a circuit for emotion, as proposed by James Papez.

At the invitation of Pat Wall, O’Keefe next moved to University College London and into the Department of Anatomy and Developmental Biology, headed by J.Z. Young—another pioneer in relating behavior to neuronal activity (in this case, in the squid). There he began to look at the hippocampus in freely moving rodents as they foraged for scattered food reward. The initial paper, written with John Dostrovsky (an MSc student passing through the lab on the way to becoming a neurologist), describes 8 out of 76 putative neurons responding to the location of the animal, with other neurons responding to arousal, attention, or movement, or responding in inconsistent or uninterpretable ways (O’Keefe and Dostrovsky, 1971). The responses of many of these cells were consistent with similar early findings by Jim Ranck.

**Figure 1:**
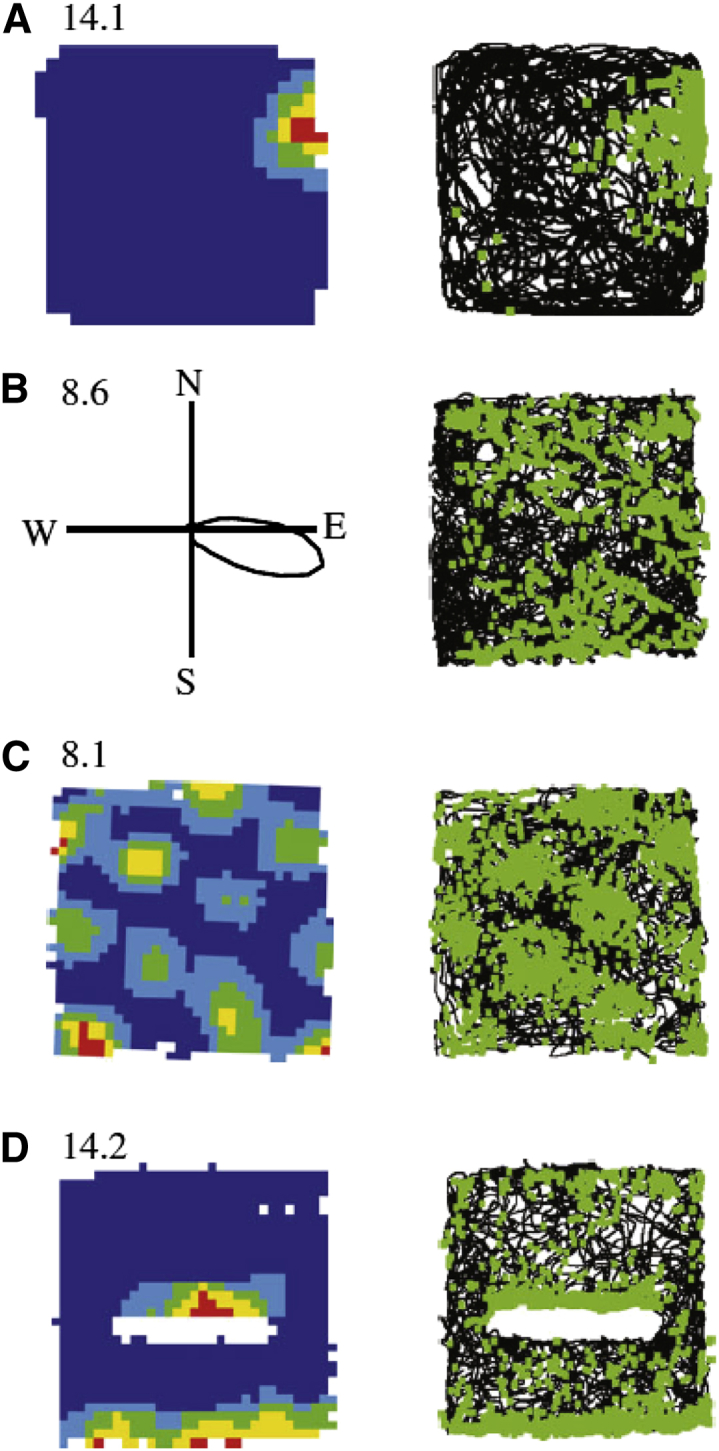
Examples of the Four Main Types of Spatial Firing Recorded from Neurons in the Hippocampal Formation of Freely Moving Rats Each example shows firing rate as a function of location or head direction (left; peak firing rate in Hz above) alongside a plot of the rat’s path within a square box (black line) and the location of the rat when an action potential was fired (green dots; right). (A) Place cells, found in areas CA3 and CA1 of the hippocampus proper, typically fire in a restricted portion of the environment. (B) Head-direction cells, found in the presubiculum and deep layers of medial entorhinal cortex, typically fire for a narrow range of allocentric heading directions (left). (C) Grid cells, found in medial entorhinal cortex and pre- and parasubiculum, typically fire in a regular triangular array of locations. Directional grid cells or “conjunctive” cells are also found, whose grid-like spatial firing is also modulated by head direction. (D) Boundary cells, found in subiculum and entorhinal cortex, typically fire at a specific distance from an environmental boundary along a specific allocentric direction. A 62 × 62 cm box was used for (A) and a 1 × 1m box for (B)–(D), with a 50 cm barrier within the box for (D); successively hotter colors show quintiles of firing rate, and unvisited locations are shown in white. Adapted with permission from Hartley et al. (2014).

Perhaps surprisingly, given the apparently weak initial support, O’Keefe immediately recognized these neurons as relevant to a classic question in cognitive psychology—that of how you represent your own location relative to the environment—and christened them “place cells.” In fact, this strong interpretation was aided by the last seven cells recorded all being place cells—indicating to O’Keefe that they were the dominant response, once the electrodes had reached the “right” brain location. Subsequent experiments revealed that a majority of the principal cells in regions CA1 and CA3 of the hippocampus that are active in a given environment are place cells, firing action potentials at a high rate whenever the animal enters a small portion of its environment and remaining silent elsewhere (see Figure 1A).

Inspired by the discovery of place cells, O’Keefe and Nadel wrote *The Hippocampus as a Cognitive Map*. This was a spectacular tour de force, specifically concerning the likely functional role of the hippocampus, but also providing a revolutionary manifesto for how to think about brain function, from the deep analysis of the function in question (representation of space) through extensive review of behavioral, lesion, and electrophysiological data to potential neuronal mechanisms and broader implications for learning and memory in humans. They concluded that there must be something like an innate unitary spatial framework onto which experience of the world can be mapped, as foreshadowed by the philosopher Immanuel Kant, and that its implementation in the brain should provide the animal with a “cognitive map” in the sense argued for by Edward Tolman: that is, an abstract and flexible representation of space allowing novel inferences (e.g., directions of travel), beyond simple representations of specific sensory stimuli, bodily responses, or associations between them. Within this framework, the place cells were held to represent the animal’s current environmental location.

The initial place cell findings aroused great skepticism, both regarding the basic finding and potential confounds such as uncontrolled local sensory stimuli (e.g., odours), and the bold and highly abstract interpretation (cognitive maps) as opposed to simpler well-studied forms of learning such as conditioned responses. These justifiable queries were duly addressed, in sophisticated form. O’Keefe first showed that the orientation of the firing locations (“fields”) could be controlled by the constellation of distal cues (O’Keefe and Conway, 1978) and then that their firing was also robust to the subsequent removal of the controlling cues (O’Keefe and Speakman, 1987). In the absence of controlling cues, the orientation of the location of a place cell’s firing within the environment was unpredictable but remained consistent with those of other place cells and with the behavioral choices of the animal. In addition, O’Keefe laid out his proposal that place-cell firing must reflect an internally generated spatial signal based on path integration in combination with environmental sensory inputs (O’Keefe, 1976), which is considered further in the next section.

The firing pattern of place cells is specific to a given environment; place cells may fire in one environment but not fire, or fire with a different spatial pattern, in another (O’Keefe and Conway, 1978; Muller and Kubie, 1987)—a phenomenon known as “remapping.” The firing rate of place cells can be modulated by other factors, such as the presence or absence of particular sensory stimuli (O’Keefe, 1976) and aspects of behavior such as running speed and direction, but primarily reflects the animal’s location within an environment. Detailed quantification of the relationship between place-cell firing and the surrounding environment came from the New York group of Bob Muller, John Kubie and Jim Ranck. Other groups followed up the nonspatial modulation of firing, and Neal Cohen and Howard Eichenbaum produced an account of hippocampal function generalizing the flexible spatial learning stressed by O’Keefe and Nadel to all forms of flexible relational learning. Further technological advances in isolating the electrical signals from single neurons came with the development of the stereotrode with Bruce McNaughton and Carol Barnes during their visit to the O’Keefe lab, and the tetrode with Michael Recce. The power of multielectrode recording was demonstrated by Matt Wilson and Bruce McNaughton recording enough place cells simultaneously to be able to accurately reconstruct the location of the rat.

Evidence for a causal relationship between the hippocampus and the specific aspects of spatial memory that might require a cognitive map came from sophisticated tests of the effects of hippocampal lesions. The most prominent of these is the Morris water maze, in which the animal must find a goal location (a platform hidden under the surface of a pool of opaque water) indicated by distal cues rather than any local sensory cues, starting from different locations around the edge of the pool. Performance in this task was shown to be impaired by hippocampal lesions, in a collaboration between O’Keefe and Richard Morris.

### Grid Cells and Other Spatial Cells

The identification of place cell firing as representing location within a cognitive map cried out for the neural support of other elements necessary for a complete navigational system: a representation of direction and a distance metric. O’Keefe and Nadel had predicted that directional signals were available to the hippocampus, and speculated that theta might signal spatial translation. The first of these to be found was the directional system, with the discovery of “head-direction cells” by Ranck and the New York lab. These cells, initially identified in the presubiculum but later found throughout Papez’ circuit, complemented place cells, each firing whenever the rat faced in a certain direction, irrespective of its location (see Figure 1B). Different cells have different preferred firing directions that are strongly controlled by distal visual cues, if present. There is a robust intrinsic organization to the population activity, such that all preferred firing directions rotate together coherently with each other. Further work, continued by Jeff Taube, showed that these neurons appear to underlie our sense of direction and also provide the directional reference for other spatial representations in the hippocampal formation (Taube, 1998).

Around this time, Edvard and May-Britt Moser were beginning their PhDs in the lab of Per Andersen in Oslo. As well as working on the neural mechanisms of learning, they became interested in the relative contributions of dorsal and ventral hippocampus to spatial memory, in collaboration with Richard Morris. Performing local “minislab” lesions at different dorsoventral levels of the hippocampus, with the anatomical precision that would become a hallmark of their work, they showed the increasing reliance of spatial memory on dorsal rather than ventral hippocampus (Moser et al., 1995). Expanding their interests to electrophysiology, they first visited John O’Keefe’s lab and then that of Bruce McNaughton and Carol Barnes to become proficient in recording from the hippocampus of freely moving rodents.

The Mosers’ interest in anatomy and the dorsoventral distinction in the hippocampus led them to aim their tetrodes at the dorsal part of the medial entorhinal cortex (mEC). Here, aided by the anatomical knowledge of Menno Witter, they hoped to find the most strongly spatial inputs to the hippocampus, because the medial (cf. lateral) entorhinal cortex receives projections from the presubiculum, where head-direction cells are found, and the dorsal portion of mEC projects to the most spatial, i.e., dorsal, portion of the hippocampus. There, with Marianne Fyhn and colleagues in their lab in Trondheim, they found neurons with multipeaked spatially modulated firing (Fyhn et al., 2004), revising earlier findings of simple spatially modulated responses in more ventral portions of mEC. There were signs that the firing peaks might be regularly arranged, but this was not clear given the small numbers of peaks present within the standard-sized box used for recordings. The final piece of the puzzle was supplied by Bill Skaggs, working with Bruce McNaughton, who suggested that they try recording in larger boxes. This simple but crucial insight produced immediate dividends. With Torkel Hafting and colleagues in their lab, they found that grid cells in dorsomedial EC fired with fantastically regular precision at the vertices of an equilateral triangular grid arranged across the environment, despite the highly irregular foraging movements of the rats (Hafting et al., 2005) (see Figure 1C).

The firing patterns of nearby grid cells have similar orientation and scale but vary from each other in terms of their spatial offset. The orientation of the grid pattern reflects external environmental cues, similarly to place cells, and is also probably determined by head-direction cells. The grid firing patterns recorded in dorsal locations have the smallest spatial scale, with increasingly larger grids recorded more ventrally (Hafting et al., 2005). Interestingly, the grid scale appears to be quantized (Barry et al., 2007), increasing dorsoventrally in discrete jumps (Stensola et al., 2012). The orientations of the grid firing patterns of different scale, while less tightly related than those of the same scale, also tend to cluster around similar values (Barry et al., 2007; Stensola et al., 2012). Unlike place cells, whose firing patterns can remap completely between different environments, grid cell firing patterns are basically maintained across all environments visited by the animal, with only their spatial offset to the environment varying (Fyhn et al., 2007).

Grid cells, head-direction cells, and place cells are part of a wider spatial system. Grid cells were initially described in layer II of mEC, where they are most densely represented, but were also found in the deeper cell layers of mEC and in the neighboring pre- and parasubiculum, by Francesca Sargolini and Charlotte Boccara in the Moser lab. In these other locations, directionally modulated grid cells, or “conjunctive” cells, were also found. The firing of these cells shows the grid-like spatial modulation characteristic of grid cells but is also modulated by the head direction of the animal (like head-direction cells, but typically with broader directional tuning).

To summarize, grid cells show precisely regularly distributed spatial firing patterns which are broadly invariant to environmental change and are organized (across cells) into an almost crystalline structure of quantized scales and orientations. Thus, like place cells, grid cells seem to represent an abstract sense of location rather than specific local sensory cues. However, grid cells go far beyond place cells in representing an abstract spatial framework, providing the most striking embodiment of internal cognitive structure being applied to the external world.

Environmental information must play a part in creating firing patterns that are spatially stable in the external word, and the way in which this information is represented has begun to be understood in recent decades. As noted above, place cells “remap” (i.e., fire in unrelated fashion) in distinct environments, but more subtle parametric changes to an environment’s shape and size can produce corresponding parametric changes in place-cell firing patterns (O’Keefe and Burgess, 1996), indicating the presence of inputs tuned to the distance and allocentric direction of environmental boundaries. The proposed input neurons (“boundary vector cells” or “border cells”) were subsequently found in the subiculum and entorhinal cortex, reviewed in Hartley et al. (2014) (see Figure 1D).

In search of the mechanism by which place-cell firing can vary in rate, the field turned to investigate lateral EC, led by the Mosers and also the lab of Jim Knierim. Significant environmental change causes place-cell remapping, and this is accompanied by shifts in the grid patterns in medial EC relative to the environment (Fyhn et al., 2007). However, less significant environmental changes can produce changes in place-cell firing rate only (“rate remapping”), which are not accompanied by shifts in the grid patterns (Fyhn et al., 2007). With Li Lu, the Mosers showed that this rate remapping depends on input from the lateral EC. Changes in place-cell firing rates can also reflect the presence or absence of local sensory cues (O’Keefe, 1976), and the Moser and Knierim labs have since discovered lateral EC neurons that respond to local sensory cues, the presence of objects, and even the absence of recently present objects.

The stage is set: the work of John O’Keefe, Edvard and May-Britt Moser, and their colleagues and collaborators over the years has identified the neural representations comprising a spatial navigation system in the mammalian brain. From this foundation of plentiful knowledge we can begin to ask how these representations work together to underlie behavior, learning, and memory—an exciting challenge for systems neuroscience in the years to come.

### Far-Reaching Implications of the Study of Hippocampal Spatial Coding

The work of the Nobel laureates has outlined the neural mechanisms supporting navigation, an immense achievement in itself. But the importance of their findings can been seen even more clearly when considering the profound implications they have had for our understanding of the neuronal mechanisms for representation, learning, and memory more generally. The hippocampus and spatial navigation has proved an ideal model system for understanding the relationship between brain and behavior and the principals of neural coding.

#### Implications for Intrinsically Structured Neural Representations

Grid cell firing provides a spectacular example of internally generated structure, both individually and in the almost crystalline organization of the firing patterns of different grid cells. A similarly strong organization is seen in the relative tuning of head-direction cells. This strong internal structure is reminiscent of Kantian ideas regarding the necessity of an innate spatial structure with which to understand the spatial organization of the world. To investigate the experience dependence of the spatial firing patterns, both O’Keefe and Moser groups recorded from hippocampus and medial EC in preweanling rat pups as they first began to explore outside the nest. Intriguingly, head-direction firing was present at adult levels from the first recordings, and place-cell firing was also present, but improved with further experience, while grid cell firing was not present until after several days of experience (Wills et al., 2010; Langston et al., 2010). Thus head-direction cell firing may represent a fundamental representation on which other spatial representations can be built, while place-cell firing appears not to require grid cell firing. It is possible that both environmental inputs (such as boundary vector cells) and inputs from grid cells (which may predominantly reflect path integration, see below) provide parallel routes to driving place-cell firing, so that either is sufficient and both can be combined when present.

The strong internal consistency of head-direction cell firing suggests that the dynamics of their population activity is best described as a line attractor. That is, symmetrical recurrent connectivity constrains population activity to coherent patterns representing a single head direction and allows smooth transitions between them (Zhang, 1996). The addition of asymmetric interactions dependent on the animal’s angular velocity could enable active representation of head direction. Two-dimensional attractor models were then applied to place-cell firing by Bruce McNaughton and others, with asymmetric connectivity between velocity-modulated place representations resulting in accurate path integration. These models were even more appropriate for grid cell firing, whose repeating firing patterns could reflect recurrent interactions or “Turing patterns” (reviewed by McNaughton et al., 2006). In this case, asymmetric connectivity between velocity modulated grid representations enables accurate path integration—that is, updating of an internal position estimate on the basis of self-motion cues. As with head-direction cells, the strong relative coherence of grid cell firing offers support for the symmetrical interactions posited by these models. Taken together, these findings provide strong support for the general coding principal of continuous attractor dynamics in populations of locally tuned neurons.

Perhaps the most intriguing aspect of the structure manifested by grid cell firing is its obvious potential power as a coding scheme—almost as if it were designed by a mathematician or engineer. The grid cells are organized into modules with different spatial scales (Barry et al., 2007; Stensola et al., 2012), so that each module provides a modulo code for location with modulus equal to the grid scale. Together a relatively small number of modules have a potentially exponential coding capacity representing a neural code of unprecedented power and efficiency (e.g., Sreenivasan and Fiete, 2011). How this code is actually used in the brain remains a question of intense interest and may depend critically on whether grid cells can provide a regular grid-like representation over large-scale space, given that grid regularity and scale can be strongly affected by the structure of the environment (Derdikman et al., 2009; Barry et al., 2007).

#### Implications for Oscillatory Processing and Temporal Coding

Following earlier work by Case Vanderwolf on the 4–10 Hz theta rhythm of the local field potential (LFP), and the observation of theta-modulated neuronal firing in the hippocampus by Steve Fox and Ranck, O’Keefe and Nadel assumed that it reflected a key role of the hippocampus in linking perception to action (specifically translational motion). Careful consideration of the timing with which place cells fire bursts of spikes as the animal runs through the firing field led O’Keefe and Michael Recce to realize that the phase of firing relative to the LFP theta rhythm encoded distance traveled through the firing field (O’Keefe and Recce, 1993), with the place cell firing at successively earlier phases of theta as the animal moves through the firing field. This finding represents one of the most robust examples of internally generated temporal or phase coding of a behavioral variable in neuroscience. The consequence of “phase precession” in individual place cells is that within each theta cycle the location represented by the population of active cells sweeps forward, potentially contributing to route planning at decision points, as noted by David Redish.

O’Keefe and Recce (1993) suggested that phase precession results from the interference of two oscillators—one reflected in the LFP and another with a slightly higher frequency that should increase with running speed. Theta phase precession is also observed in grid cells (Hafting et al., 2008), whose periodic spatial firing is more naturally described as an interference pattern. The idea that speed could be coded as a frequency difference, such that phase differences represent distance traveled, provides a parsimonious mechanism for path integration whose output could be the grid cell firing patterns. Such a mechanism is broadly supported by relationships between running speed, grid scale and the frequencies of subthreshold membrane potential oscillations, LFP theta and burst firing by grid cells, and by “theta cells,” and by the elimination of grid patterns by disruption of the theta rhythm (reviewed in Burgess and O’Keefe 2011), and would complement the stability provided by attractor dynamics. However, the interpretation of the role of theta rhythmicity remains hotly contested, given only mixed support from analyses of grid scale and firing rhythmicity in the Moser lab, the strong relationship between grid cell firing and tonic depolarization, and the existence of grid cell in bats in the absence of theta.

The interaction between theta and gamma oscillations opens a window into studying interregional communication and mode-switching within the hippocampal formation. Gamma is a broad higher-frequency band (25–140 Hz) associated with local inhibitory mechanisms that is prevalent in neocortical areas but also visible within the hippocampal formation. Following work from Gyuri Buzsaki’s lab linking the generation of low-frequency (25–50 Hz) gamma oscillations to the CA3 region of the hippocampus and higher-frequency gamma oscillations to the entorhinal cortex, the Mosers and Laura Colgin began to investigate whether gamma might shed light on interregional communication with region CA1. They found that periods of high- or low-frequency gamma in the CA1 local field potential were indicative of periods of place cell firing being driven by EC or CA3, respectively, with each gamma band associated with different phases of theta. Thus, different gamma frequencies might allow flexible routing of information from distinct inputs to CA1, extending the idea of interregional communication via gamma band coherence suggested by Wolf Singer and Pascal Fries, and consistent with Mike Hasselmo’s suggestion of encoding (entorhinal-driven) versus retrieval (CA3-driven) scheduling by theta phase.

#### Implications for Memory

Scoville and Milner’s (1957) identification of the hippocampus as a critical structure for supporting memory and the observation of dense recurrent connectivity in region CA3 inspired David Marr (Marr, 1971) to produce the canonical model of hippocampal memory. Recordings from the hippocampus of freely moving rats provided a test bed, verifying many of the main predictions, including the presence of attractor dynamics and pattern completion in place-cell firing.

One intriguing aspect of the Marr model is that the rapidly stored information in the hippocampus is transferred to long-term storage in the neocortex. Both Gyuri Buzsaki and Bruce McNaughton explored the implications of this suggestion. With Matt Wilson, McNaughton searched for the recapitulation of place-cell firing patterns from a day’s experience during subsequent sleep. Encouragingly, they found “replay” of firing sequences from active exploration during subsequent slow-wave sleep (Wilson and McNaughton, 1994). Buzsaki focused on “ripples” as the most likely means of communication from hippocampus to neocortex. These short bursts of high-frequency activity in the hippocampal LFP, first identified by O’Keefe and Nadel (1978), co-occur with “replay” events during slow-wave sleep and during wakefulness. Interestingly, the disruption of ripples immediately after learning disrupts subsequent spatial memory performance, consistent with a role in the consolidation of hippocampal information to neocortex.

*The Hippocampus as a Cognitive Map* by O’Keefe and Nadel (1978) also prompted a new perspective on hippocampal function in human memory—specifically relating hippocampal amnesia (Scoville and Milner, 1957) to the idea of context-dependent or “episodic” memory introduced by Endel Tulving. The place cells could provide a substrate for spatial context, to which O’Keefe and Nadel argued that temporal context would be added in humans, with the role of context in learning being further developed by Nadel. One specific suggestion, arising from work with O’Keefe, is that the various spatial firing properties of neurons within Papez’ circuit provide the spatial structure within which the contents of memory can be imaged (Burgess et al., 2001). This idea received recent support in the observation that the spatial coherence of imagery as impaired in hippocampal amnesics (Hassabis et al., 2007). However, the role of the hippocampus in human memory remains controversial to this day. Some authors, including Nadel and Morris Moscovitch, argue for a specific role in episodic or contextual memory and question the extent and nature of consolidation to other areas. Others, most notably Larry Squire, hold to a time-limited but more general role in explicit or declarative memory, in line with earlier definitions of amnesia.

Within the spatial domain, the discoveries of place cells and grid cells in rodents have given rise to several related findings in humans. The activity and structural integrity of the human hippocampal formation has been related to accurate spatial navigation (e.g., reviewed in Burgess et al., 2002), with these experiments often making use of virtual reality adaptations of hippocampal-dependent tasks used with rodents, or an analog of comparative neuroethology in the study of taxi drivers by Eleanor Maguire. Evidence for neurons with firing resembling place cells and grid cells has come from intracranial recordings in epilepsy patients by the labs of Mike Kahana and Izhak Fried. Intriguingly, the wide distribution of locations containing grid-cell-like activity suggests that they may play a more general (nonspatial) role in autobiographical memory.

As with spatial navigation, the work of the 2014 Nobel laureates has provided a strong foundation upon which a mechanistic, neural-level understanding of memory can be built. Knowledge of the relevant neural representations becomes even more powerful in combination with an understanding of how changes in synaptic connectivity between neurons can mediate learning via long-term potentiation, a phenomenon that was also discovered in the hippocampus by Tim Bliss and colleagues. Further advances, such as the application of molecular biology and optogenetics to manipulate hippocampal representations, are beginning to allow investigations and manipulations of memory that would previously have been considered unreachable (e.g., work by Susumu Tonegawa, recipient of the Nobel Prize in 1987 for work on immunology). These experiments include the deletion of fearful memories via disrupted reconsolidation and the artificial creation of memories of fear or reward.

### Conclusion

The energy, enthusiasm, and vision of John O’Keefe, Edvard Moser, May-Britt Moser, and their colleagues have left the field of cognitive neuroscience with a model system—hippocampal spatial navigation—in which much of the neuronal code has been cracked. This tremendous achievement provides firm foundations for rapid progress in many directions and will be widely recognized as truly meriting the 2014 Nobel Prize in Physiology or Medicine. There may be a long way to go in fully understanding human memory and how it can fail in neurological and psychiatric disorders, but the foundations for a bridge from molecules through neurons, synapses, and networks to behavior and symptoms have been laid.
